# Conjunctival Inverted Papilloma Progressing to Carcinoma. First Report in Horse

**DOI:** 10.3390/vetsci8060108

**Published:** 2021-06-10

**Authors:** Vito Biondi, Annamaria Passantino, Michela Pugliese, Salvatore Monti, Alessandra Sfacteria, Simona Di Pietro

**Affiliations:** Department of Veterinary Sciences, University of Messina, 98168 Messina, Italy; v.biondi@hotmail.it (V.B.); annamaria.passantino@unime.it (A.P.); michela.pugliese@unime.it (M.P.); salvatore.monti@unime.it (S.M.); simona.dipietro@unime.it (S.D.P.)

**Keywords:** horse, eye, conjunctiva, neoplasia, inverted papilloma, squamous cell carcinoma

## Abstract

A five-year-old, entire female Arabian horse with a 6-month history of a non-painful nodule on the conjunctiva of the right eye was evaluated. Ophthalmological examination showed a firm, smooth and fleshy conjunctival mass that raised the suspicion of a conjunctival neoplasm. Histological evaluations showed that the mass was composed of an endophytic growth consisting of numerous long papillary projections of hyperplastic stratified squamous epithelium supported by thin fibrovascular stalks. Typical features of squamous cell carcinoma with disorganized cell growth and infiltration of surrounding tissues were detectable within the mass. Inverted papilloma progressing to carcinoma was diagnosed. Follow-up examination showed that no local recurrence was present during the 12-month follow-up period. To the authors’ knowledge, this is the first report describing the inverted papilloma in the horse and, due to its progression to squamous cell carcinoma, warns about the inclusion of the inverted papilloma in the differential diagnosis of conjunctival neoplasm and driven treatments.

## 1. Introduction

Papillomas or warts are common cutaneous tumors of young horses ranging from 12 to 24 months of age that recognize in their pathogenesis either equine papillomavirus or noninfectious irritants [1,2]. Only the variety caused by the viral action is referred to as papillomatosis. Papillomas are slow-growing masses that frequently affect the muzzle, eyelids, and ears. Papillomas of the ocular region represent 7% of equine ocular neoplasms [3]. Grossly, they present as elevated horny masses with fronds grouped in clusters measuring 2 to 10 cm in diameter [2]. Equine papillomas are benign exophytic tumors, usually spontaneously regressing in 1 to 6 months, eliminating the necessity for intervention [4]. Histologically, papillomas are characterized by numerous thin fibrovascular stalks covered by hyperplastic epithelium. The growth pattern may be exophytic, with the projections extending above the surface of the skin, or endophytic (inverted), with the projections extending into the dermis and hypodermis [5]. Inverted papilloma is an uncommon, benign endophytic proliferation of the epidermis; it has only been reported in dogs and humans [6]. In dogs, endophytic lesions are most commonly found on the forelimbs and abdomen as solitary lesions located within the dermis, yet have not been described so far in the conjunctiva even though the classical, exophytic, and conjunctival papilloma can affect the canine eye. In humans, inverted papilloma is mainly found in the nasal cavity and paranasal sinuses, while the conjunctival one is exceedingly rare, more than ever if associated with squamous cell carcinoma (SCC) [7].

## 2. Materials and Methods

A five-year-old, entire female Arabian horse (400 kg bwt) presented a conjunctival mass of approximately 2 cm located on the lower eyelid conjunctiva of the right eye (RE).

The owner reported a watery discharge and the presence of an enlarging mass over the last six months. The animal was active, and had been treated with tobramycin eye drops for a month without significant improvement.

Ophthalmological examination of both eyes with a transilluminator (Heine, Gilching, Germany), a portable handheld biomicroscope (Portable Slit Lamp SL-14, Kowa, Osaka, Japan), and a direct ophthalmoscope (Heine BETA 200^®^, Heine, Gilching, Germany) was performed. Tear production (Schirmer’s tear test—Tear Strips, Biovision Limited, Dunstable, UK) and intraocular pressure (IOP) (TonoPen^®^ XL, Reichert Technologies, Depew, NY, USA) were also measured.

Differential diagnoses of the mass included primary tumor and non-neoplastic lesions such as granuloma.

The owner agreed to remove the growth surgically and signed the written consent.

Before surgery, the animal underwent a complete general physical examination, which was normal, and laboratory tests, including complete blood counts (CBC) and serum biochemical profile (total protein, albumin, urea, creatinine, total bilirubin, aspartate aminotransferase, and aspartate aminotransferase) that were within the physiological ranges. The animal was pre-medicated with acepromazine (Prequillan^®^, Fatro Industria Farmaceutica Veterinaria S.p.A., Bologna, Italy) at a dose of 0.03 mg/kg intravenously. Sedation was induced using detomidine (Demosedan^®^, Vétoquinol Italia S.r.l., Bertinoro, Italy) 0.03 mg kg^−1^ and butorphanol (Dolorex^®^, MSD Animal Health S.r.l., Milan, Italy) 0.01 mg kg^−1^ intravenous administration.

The eye and associated structures instilling 0.4% oxybuprocaine hydrochloride (Benoxinato Cl^®^, Alfa Intes S.r.l., Casoria, Italy) ophthalmic drops were desensitized.

A retrobulbar nerve block using 5 mL of 2.0% lidocaine (Lidocaine 2%, Ecuphar Italia S.r.l., Milan, Italy) injected into the retrobulbar space was obtained.

The mass was successfully excised, and the animal was standing calm without any signs of pain.

The mass was sent to the pathology service of the Veterinary Teaching Hospital of the University of Messina; formalin-fixed and paraffin-embedded (FFPE) specimens were routinely cut and stained with H&E for histopathological evaluation.

The slides, prepared as above, were observed under an optical microscope (DMI6000, Leica Microsystems, Wetzlar, Germany) connected to a camera and image analysis software (Leica Application Suite X, Leica Microsystems, Wetzlar, Germany). A numerical count of mitoses was determined and reported for 2.37 mm^2^ area at high power field (400×).

## 3. Results

### 3.1. Ophthalmological Finding

**Figure 1:**
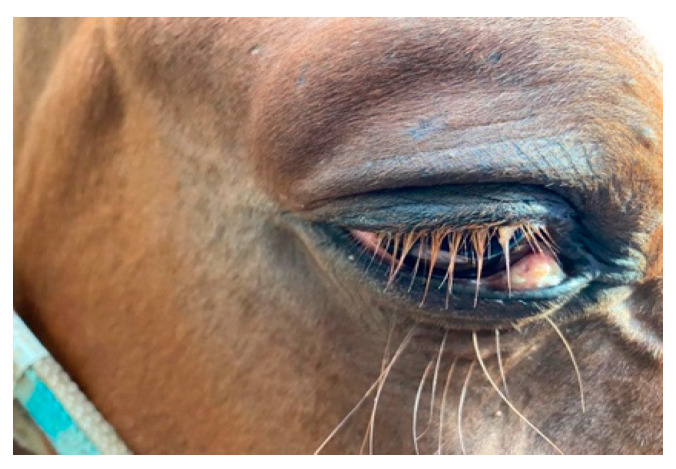
Mass on the right eye, of approximately 2 cm, located on the lower eyelid conjunctiva close to the medial canthus.

Slit lamp biomicroscopy showed in RE third eyelid and conjunctiva moderately hyperemic and a greyish white mass smooth and regular, and raised above the surrounding tissue. The conjunctival mass was approximately 2 cm in size and localized close to the medial (nasal) canthus of the lower eyelid conjunctiva (Figure 1).

The growth was also firm and moderately friable. The anterior chamber, the iris, the vitreous, and the fundus were normal.

Schirmer tear test were 18 and 27 mm/min RE and left eye (LE), respectively. IOP was found to be 25 and 23 mmHg RE and LE, respectively.

**Figure 2:**
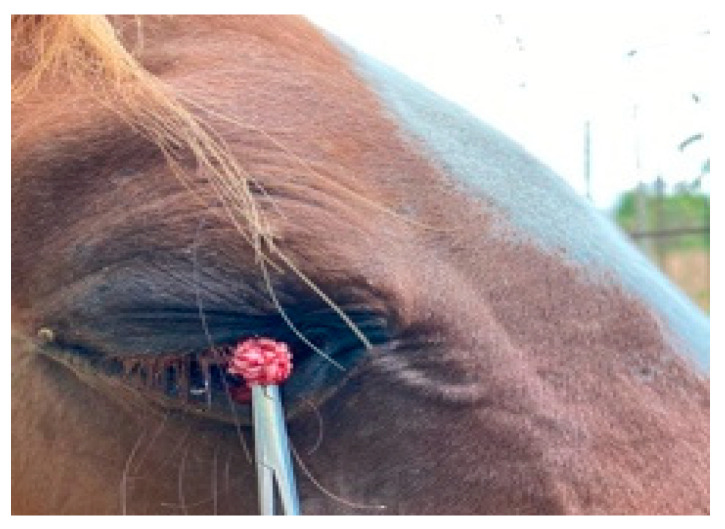
Acuminate, horny fronds at the inner surface of the conjunctival mass after surgery.

Under general anaesthesia, the nodule was excised. As soon as the mass was clamped, it went completely away, showing an inner surface covered by filiform, acuminate, and horny fronds (Figure 2).

The nodule did not invade the cornea and was approximately 2 cm in diameter. After surgery, a topical medication was performed using an eye ointment 10 mg/g chlortetracycline hydrochloride (Ophtocycline, Dechra Veterinary Products S.r.l., Torino, Italy), four times a day for 15 days. After the treatment, the animal was followed up every 2 months during the first year to rule out recurrences. Any sign of ocular recurrence was not revealed.

### 3.2. Histological Finding

**Figure 3:**
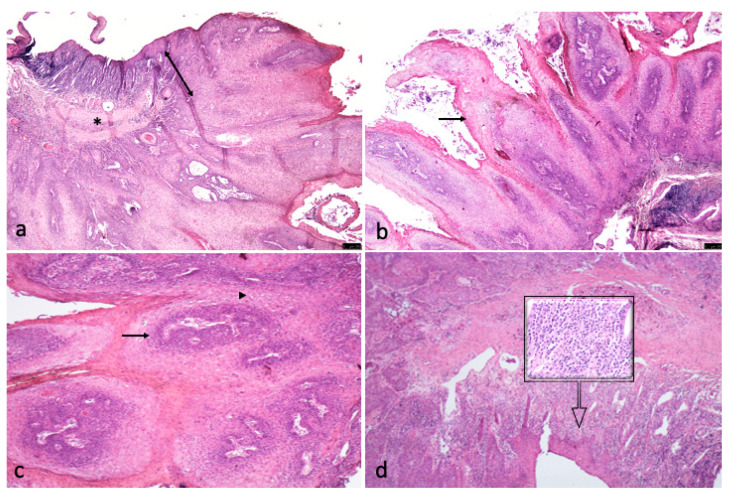
(**a**) Conjunctival epithelium (double-edged arrow) invaginating downward the underlying submucosa (asterisk, HE 5×); to form (**b**) numerous long papillary projections of hyperplastic stratified squamous epithelium (arrow, HE 5×); (**c**) cross section of papillary fronds showing a fibrovascular core surrounded by hyperplastic stratified squamous epithelium with conserved normal architecture (arrow, HE 10×). Many koilocytes were identifiable (arrowhead) (**d**) a mixed chronic inflammatory cell infiltrate, mainly composed by lymphocytes and plasma cells, was interposed between the boundaries of the lesion and the surface epithelium (HE 2.5×. Inset. 40×).

**Figure 4:**
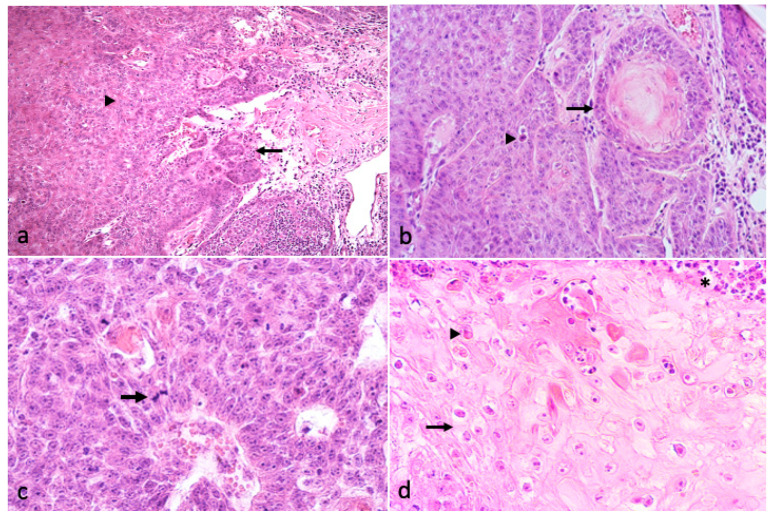
(**a**) Areas of the tumor showing a disorganized appearance and trabecular, papillar, or nest growth patterns (arrowhead). Papillary projections and nests of SCC invading the submucosa (arrow) (HE, 2.5×); (**b**–**d**) SCC cells showing mild to moderate pleomorphism, dyskeratinization, and keratin pearl formation ((**b**), arrow. 20×), acantholytic cells ((**b**), arrowhead), increased mitotic count ((**c**), arrow. HE 40×), koilocytes ((**d**), arrow), intranuclear inclusions ((**d**), arrowhead), and foci of neutrophilic inflammation ((**d**), asterisk. HE, 40×).

Histologically, conjunctival epithelium invaginated downward the underlying submucosa giving rise to numerous long papillary projections of hyperplastic stratified squamous epithelium, supported by thin fibrovascular stalks. Foci of a mixed chronic inflammatory cell, mainly composed of lymphocytes and plasma cells, were located at the periphery of the lesion and underneath the surface epithelium. The stratum basale laying to the fibrovascular stalks was hyperplastic, with a moderately high mitotic count (2–5/high power field). A prominent hyperkeratosis and keratohyalin granules characterized the proliferation. There was a regular epithelial maturation sequence even though many cells in the stratum granulosum were swollen with centrally placed nuclei often containing not well-defined, circular, basophilic structures surrounded by a clear halo and marginated chromatin being suggestive of koilocytes. Acanthocytes were also admixed within cells of normal appearance. These patterns were compatible with the cytopathic effects and the inclusions induced by papillomaviruses as already described in other equine papillomatous lesions (Figure 3 and Figure 4).

In focal areas of the mass, the growth appeared disorganized, with loss of polarity of the keratinocytes and loss of normal keratinocyte maturation invading and infiltrating the submucosal tissues. This neoplastic growth was often arranged in trabecular, papillar, or nest patterns with cells showing mild to moderate pleomorphism, multiple nucleoli, dyskeratinization, increased mitotic count (4 up to 9/high power field) and atypical mitotic figures. Some “keratinic pearls” were identifiable as well as foci of neutrophilic inflammation surrounding the carcinomatous growth. Acantholytic cells, koilocytes and cells bearing intranuclear eosinophilic inclusions were also visible at the boundaries of the malignant neoplastic growth or intermingled with the cancerous cells (Figure 4).

## 4. Discussion

In the horse, the eye can be the site of primary and metastatic neoplasms; squamous carcinoma in the limbic bulbar conjunctiva, the third eyelid, and the other eyelids being these sites directly exposed to ultraviolet radiation, are the most frequently occurring and concerning [3]. Precancerous epithelial changes as squamous plaque, squamous papilloma, squamous carcinoma in situ can temporally precede the onset of the neoplasm [8]. Squamous cell carcinoma progression from equine penile and preputial papilloma is frequent [9]. As for the classical pattern of papilloma, inverted papilloma is a benign neoplasm that can be associated with squamous cell carcinoma. In human patients, the occurrence of malignancy in all patients with inverted papilloma accounts for 9.1% [10]. Although this association is likely to occur, the exact relationship is unclear. Squamous cell carcinoma may present in the setting of inverted papilloma in three different circumstances. It has been reported that small foci of squamous cell carcinoma within inverted papilloma may differentiate; the malignancy may present as a separate synchronous lesion and not within inverted papilloma; and finally, metachronous carcinoma in areas of prior resection of benign inverted papilloma may rise [11,12,13].

The association between papillomavirus and the onset of epithelial neoplasms is well known; the papilloma resulting from the viral action is described based on the frond and cytopathic patterns other than the viral particles recognition by the mean of ancillary techniques [14,15]. In the present case, the macroscopic and microscopic appearance was suspicious of a viral etiology, although lacking molecular confirmation.

The progression from papilloma to squamous cell carcinoma has been recently addressed as an emerging concern in canine oral papilloma [16]; the association between inverted papilloma and squamous cell carcinoma has not yet been reported. In the current paper, the association with malignancy, along with the malicious growth of inverted papilloma, recommends seeking it even in other species prone to develop papilloma. This case report, describing such evidence in a horse, warns about the need to include the inverted papilloma in the differential diagnosis of conjunctival neoplasm and drives the treatment paradigm for papilloma.
